# Comparison among Different Gilthead Sea Bream (*Sparus aurata*) Farming Systems: Activity of Intestinal and Hepatic Enzymes and ^13^C-NMR Analysis of Lipids

**DOI:** 10.3390/nu1020291

**Published:** 2009-12-18

**Authors:** Laura Del Coco, Paride Papadia, Sandra A. De Pascali, Giorgia Bressani, Carlo Storelli, Vincenzo Zonno, Francesco Paolo Fanizzi

**Affiliations:** 1 Dipartimento di Scienze e Tecnologie Biologiche ed Ambientali, Università del Salento, Prov.le Lecce-Monteroni, 73100, Lecce, Italy; Email: laura.delcoco@gmail.com (L.D.C.); paride.papadia@unisalento.it (P.P.); sandra.depascali@unisalento.it (S.A.D.); g.bressani@fisiologia.unile.it (G.B.); carlo.storelli@unisalento.it (C.S.); 2 Consorzio Intercomunale Capo S. M. di Leuca, Tricase, 73039, Lecce, Italy; Email: vincenzo.zonno@unisalento.it

**Keywords:** gilthead sea bream (*Sparus aurata*), PUFA, rearing systems, food authenticity, nutritional physiological state, ^ 13^C NMR profiling

## Abstract

In order to evaluate differences in general health and nutritional values of gilthead sea bream (*Sparus aurata*), the effects of semi-intensive, land-based tanks and sea-cages intensive rearing systems were investigated, and results compared with captured wild fish. The physiological state was determined by measuring the activity of three different intestinal digestive enzymes: alkaline phosphatase (ALP), leucine aminopeptidase (LAP) and maltase; and the activity of the hepatic ALP. Also, the hepatic content in protein, cholesterol, and lipid were assessed. ^13^C-NMR analysis for qualitative and quantitative characterization of the lipid fraction extracted from fish muscles for semi-intensive and land based tanks intensive systems was performed. The lipid fraction composition showed small but significant differences in the monounsaturated/saturated fatty acid ratio, with the semi-intensive characterized by higher monounsaturated and lower saturated fatty acid content with respect to land based tanks intensive rearing system.

## 1. Introduction

Gilthead sea bream is one of the most important finfish species cultured in the Mediterranean region and its production is still in rapid expansion [1]. Despite production of these species having reached a high level of quality and efficiency, the knowledge of its nutritional requirements and digestive processes is very scarce compared to other fish species [2]. In recent years there has been an increase in demand for seafood products in our country. Therefore, both fishing efforts (with consequent fish resource impoverishment and aquaculture development), and fish importations from other countries have became the object of monitoring and investigation. Compared to meat, seafood consumption is lower, notwithstanding its nutritional properties [1]. First of all, fatty acids contained in fish, in particular eicosapentaenoic acid (20:5 *n*-3 or EPA), have important health functions for the body and are different from those of beef and pork, the latter being rich in cholesterol. On the contrary, seafood fatty acids have the ability to a decrease cholesterol blood level and are therefore very useful for the prevention of cardiovascular diseases [2].

In particular, seafood fatty acids are critical in the human diet because they are one of the more widely available natural sources of eicosapentaenoic (EPA) and docosahexaenoic (22:6 *n*-3 or DHA) acids. The dietary uptake of *n*-3 fatty acids is essential for growth and, in particular, for the health of mitochondrial and cell membranes. Indeed, *n*-3 fatty acids are involved in haemoglobin synthesis, coagulation mechanisms, prevention of capillary fragility and other processes such as reproduction. Some breast diseases and menstrual cycle alterations result from excessive uptake of saturated fatty acids and variation of *n*-3/*n*-6 ratio. Moreover *n*-3 confers a better tolerance to carbohydrates in diabetics and are precursors of prostaglandins.

Several attempts have been made to determine fatty acid composition in different types of natural fats such as fish lipids [3], milk fat [4], animal fat [5] and edible oils, in particular olive oils [6,7]. Analysis of chemical parameters was performed by using traditional methods [8,9] in association with other analytical techniques [10]. Among them, high resolution ^1^H-, ^13^C- and ^31^P-nuclear magnetic resonance (NMR) was found to be a valuable tool for the lipids analysis (including fish lipids). In addition to fatty acid composition and lipid classes, ^13^C-NMR, in particular, gives information about the regiospecific distribution of fatty acids on triacylglycerols (TAGs) [11,12] and phospholipids (^13^C- and ^31^P-NMR) [13,14,15]. Knowledge of TAGs structure has also become increasingly important since the stereospecific structure influences the lipid metabolism [16,17] and bioavailability of fatty acids.

Although fish tissue varies according to factors such as season, age, diet, and environmental factors, there are major genetic differences among species. The different chemical shifts of acyl chains in sn-1,3 and sn-2 positions allow quantitative analysis of the fatty acids distribution in TAGs by ^13^C-NMR. The carbonyl region (172–174 ppm), olefinic signals (126–134 ppm), glycerol region (60–74 ppm), and the aliphatic region (19–35 ppm) of ^13^C-NMR spectra have been used, in order to qualitatively and quantitatively evaluate the lipid fraction composition of gilthead sea bream (*Sparus aurata*), reared in different farming systems [7,11,12,18,19]. The most important share of the commercially available gilthead sea bream in the Italian market is reared under the intensive fish culture method (both in land based tanks and sea cages), according to Italian fish farmers association (Associazione Piscicoltori Italiani API, http://www.api-online.it). Three different fish rearing methods have been investigated in the present work: semi-intensive, land based tanks intensive and sea cages intensive, the first opening the way to interesting perspectives for quality control of the fish product. In addition to genetic factors, different type and mode of feeding, population density in tanks or cages, the swimming activities and other environmental factors (temperature, salinity, pH, oxygenation, *etc*.) may influence the fish organoleptic characteristics (colour, flavour) and (within a specific range) chemical composition, particularly of the lipid component.

In order to evaluate the possible differences in nutritional values, the effects of the three rearing systems on several parameters of the gastro-intestinal function, describing general health of the specimens, were investigated and compared with wild type (used as control). General similarity of the physiological state of the reared fishes with that of wild sea breams, allowed us to focus subsequent investigations on the evaluation of qualitative and quantitative composition of the lipid fraction extracted from fish muscles. In particular, saturated and essential polyunsaturated fatty acids (*n*-3 and *n*-6) of commercial size reared gilthead sea bream (*Sparus aurata*) specimens, obtained with different methods of fish culture: semi-intensive (Acquatina basin, Frigole-Lecce, Puglia, Italy) and intensive in land based tanks (Maribrin s.r.l. aquaculture system, 8 Km south of Brindisi, Puglia, Italy) were further studied by ^13^C-NMR.

## 2. Results and Discussion

The nutritional physiological state (evaluated by measuring the activity of three different intestinal digestive enzymes and flesh composition) and the NMR characterization of the lipid fractions will be discussed separately.

### 2.1. Flesh Composition and Activity of Intestinal Digestive Enzymes

The results concerning hepatic protein content indicated that only the protein amount measured in the semi-intensive reared fishes changed during the trial, being higher (803.17 ± 38.63 mg protein/g dry tissue, data not shown) at the beginning of the trial and lower at the end (377.73 ± 4.15 mg protein/g dry tissue), while it remained unchanged in the land based tanks intensive and sea-cages intensive animals. The hepatic protein content of fish reared in land based tanks was the highest at the end of the trial, whereas lipids were lower with respect to the other reared fish groups. Also, in order to evaluate the general health condition of the reared fish, data for wild sea bream captured at the end of the trial period are shown for comparison. The cholesterol content appeared to be similar in all reared fish conditions, but lower with respect to the wild type control (Table 1, Table 2).

**Figure 1:**
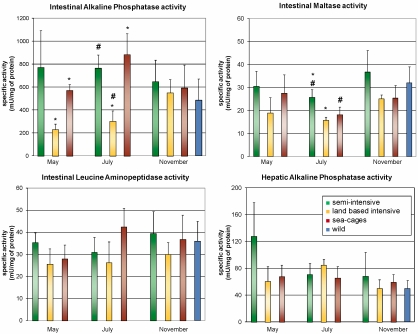
Top left, intestinal alkaline phosphatase activity; bottom, left intestinal leucine aminopeptidase activity; top right, intestinal maltase activity; bottom right hepatic phosphatase activity. Data for wild specimens, used as control, are shown only for the last month of the trial period. Each value represents mean ± SD of 3 replicates. Pairs of significantly different mean values (based on a t-test for independent samples, p-values < 0.05) are label with the same symbol. Homogeneity of variances is always satisfied at a p-value of 0.05.

Fishes reared in the semi-intensive and in the sea-cages intensive conditions showed a significantly higher intestinal alkaline phosphatase (ALP) activity during the trial, but no significant differences in ALP enzymatic activity were observed at the end of the trial for all groups investigated with respect to the control. Also, in the case of maltase activity the semi-intensive and sea cage intensive reared fish exhibited higher activity with respect to the fish reared in land based intensive condition with the difference in activity becoming negligible at the end of the trial. Concerning the activity of leucine aminopeptidase (LAP), no significant differences among groups were observed at the beginning of trial. However, at the end of it the land based tanks intensively reared fish showed a lower activity, whereas the semi-intensive and sea-cages intensive reared fish showed no significant differences between them and the control. Hepatic ALP activity did not show significant differences among groups, except for the initial value measured in the semi-intensively reared fish. Altogether, the enzymatic activities measured in reared fish at the end of the trial did not show significant differences from those measured in wild sea breams (Figure 1) at the end of the trial. Due to the similarity of the physiological state of the fishes reared in the land based and sea cages intensive regime, subsequent investigations focused on the evaluation of qualitative and quantitative composition of the lipid fraction extracted from fish muscles of only two rearing systems: the semi-intensive and the land based intensive, since the latter is the most prevalent commercial intensive rearing system (ISMEA-Istituto di Servizi per il Mercato Agricolo Alimentare, http://www.ismea.it). 

**Table 1:** Hepatic protein, lipids and cholesterol contents for the three different rearing systems, and wild samples (control) at the end of trial. Each value represents mean ± SD of 3 replicates.

(mg/g dry tissue)	Semi-intensive	Land based intensive	Sea cages	Wild sea breams
**Protein**	377.73 ± 4.15	585.88 ± 32.82	488.57 ± 15.89	486.45 ± 15.31
**Lipids**	626.00 ± 62.39	358.92 ± 30.05	414.68 ± 56.52	506.27 ± 75.44
**Cholesterol**	6.31 ± 0.76	9.1 ± 0.11	6.05 ± 0.01	11.07 ± 0.57

**Table 2:** Significance of differences (p-values) in hepatic protein, lipids and cholesterol contents for the three different rearing systems, and wild samples (control) at the end of trial, based on a t-test for 3 independent samples. Homogeneity of variances is always satisfied at a p-value of 0.05.

	Semi-intensive vs Land based intensive	Semi-intensive vs Sea Cages	Semi-intensive vs Wild Sea Breams	Land based intensive vs Sea Cages	Land based intensive vs Wild sea breams	Sea cages vs Wild sea breams
**Protein**	<0.001	0.004	<0.001	0.010	0.009	0.876
**Lipids**	0.003	0.006	0.069	0.206	0.035	0.168
**Cholesterol**	0.003	0.589	<0.001	<0.001	0.004	<0.001

### 2.2. ^13^C-NMR Spectra

^13^C-NMR spectra of the different reared samples of *Sparus aurata* (land based tanks intensive and semi-intensive at the end of trial) were processed by applying a correction of the baseline (using a polynomial function). Then, in order to facilitate integration of peaks arising from the *n*-3 fatty acids at the sn-1,3 and sn-2 positions, deconvolution was performed in the carbonyl and methyl regions of the spectra. Finally, integration of signals chosen for their significance, on the basis of literature data [20,21,22,23,24,25], was accomplished.

**Figure 2:**
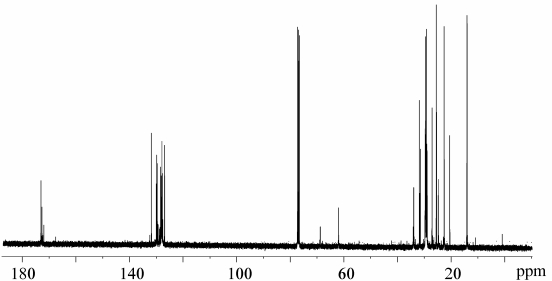
^13^C-NMR spectra in CDCl_3_ of *Sparus aurata* lipid fraction.

**Figure 3:**
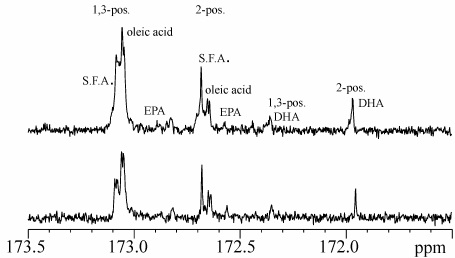
Expansions of ^13^C-NMR spectra of *Sparus aurata* lipid fractions relative to the carbonyl carbons region. 1,3-pos. and 2-pos. are referred to the position of esterified fatty acids on glycerol.(Top: intensive; bottom: semi-intensive; S. F.A.: saturated fatty acids).

In order to calculate fatty acid composition for each sample, the weighted average of signals at 62.01 ppm and 68.83 ppm, belonging respectively to α and β carbons of the glycerol moiety, was chosen for normalization as an internal standard [26]. A typical ^13^C-NMR spectrum of the gilthead sea bream lipid fraction is shown in Figure 2. The main differences among the samples from two different farming systems are reflected on intensities of signals in the ^13^C-NMR spectra. The expansion of carbonyl carbons (C1 atoms) region (173.5–172.0 ppm) is shown in Figure 3. Resonances were assigned according to previous studies on fish lipids [9]. In general, the chemical shifts of carbonyl carbons of fatty acids in triacylglycerols depend on the regiospecific position (whether the fatty acid is a sn-1,3 or sn-2 chain), and also on the position and number of double bonds in unsaturated fatty acids [27,28].

The intensities of the resonances of the acyl chains in triglycerides describe the fatty acid profile of the different samples. The chemical shifts of carbonyl carbons of the fatty acids are related to differences in the specific esterified position on the glycerol: those at high frequencies (deshielded signals) are related to fatty acids esterified in the 1,3 positions of glycerol, whereas the corresponding fatty acids in position 2 are shielded by 0.40 ppm with respect to the sn-1,3. This difference in chemical shift is always observed, regardless of the fatty acid investigated.

In the carbonyl region of the ^13^C-NMR spectra (Figure 3), signals of the saturated fatty acids (stearic acid, palmitic acid) and oleic acid are found in the same order, from high frequencies (sn-1,3 position in the glycerol moiety) to low frequencies (sn-2 in the glycerol moiety), while the peaks of linoleic and linolenic fatty acids overlap (at the used instrument field) to give a single signal (not shown). In particular, the polyunsaturated fatty acids (PUFA) of the series *n*-3 (docosahexaenoic acid, DHA, and eicosapentaenoic acid, EPA) are more shielded with respect to other fatty acids [29].

Assignments were confirmed by resonances present in the region of the vinylic carbons, where, despite the greater overlap, it is possible to distinguish the signals related to *n*-3 PUFA from the polyunsaturated fatty acids not belonging to the *n*-3 series (*n*-6 PUFA).

It should be noted that in the studied samples palmitic (16:0) and stearic (18:0) fatty acids are the most abundant among the saturated FA, while oleic acid (18:1 *n*-9) is prevalent among the monounsaturated fatty acids. On the other hand, the *n*-3 fatty acids, in particular DHA and EPA are the main constituents of the polyunsaturated fraction. In all samples, regardless of the farming systems considered, the ratio of EPA/DHA is approximately constant and about 1:2 for all the extracts. As already reported in other fish samples, in the present case the polyunsaturated fatty acids are preferentially esterified in the sn-2 position of glycerol, whereas saturated and monounsaturated fatty acids are bound at the sn-1,3 position.

**Table 3:** Percentage and standard deviations of fatty acids in lipid fractions (intensive and semi-intensive specimens), calculated by ^13^C-NMR spectroscopy. (S.F.A.: saturated fatty acids; PUFA: polyunsaturated fatty acids; n-3 HUFA: highly unsaturated fatty acids). Each value is average of 3 replicates. Significant differences in means are indicated by ‘*’ (p-value 0.004) or ‘#’ (p-value 0.02) based on a t-test for independent samples. Homogeneity of variances is always satisfied at a p-value of 0.05.

Rearing System	Intensive	Semi-Intensive
**22:6 *n*-3**	7.8 ± 2.5	6.1 ± 1.7
**20:5 *n*-3**	4.1 ± 1.6	3.6 ± 0.4
**18:3 *n*-3**	8.0 ± 2.7	9.7 ± 3.9
**18:1 *n*-9**	24.1 ± 0.6*	30.9 ± 1.9*
**S.F.A.**	39.5 ± 0.7^#^	35.5 ± 1.7^#^
**PUFA**	17.0 ± 2.3	15.6 ± 4.2
***n*-3 HUFA**	19.9 ± 1.4	19.7 ± 3.0
**ratio *n*-3/*n*-6**	1.2	1.3
**ratio 18:1 *n*-9/ S.F.A.**	1.6	1.2

The percentage composition of the lipid component was analyzed for the different farming systems. The results obtained for samples (from ^13^C-NMR data) are shown in Table 3. Both type of samples show approximately the same ratio of *n*-3 and *n*-6 fatty acids, with a slightly higher ratio in samples of *S. Aurata* belonging to the semi-intensive farming system. No significant differences were specifically observed for DHA, EPA, and 18:3 fatty acids for the two rearing systems examined. Also the PUFA content shows no significant differences. On the other hand, small, but significant, differences were evident in the monounsaturated/saturated fatty acid ratio. The land based tanks intensive rearing system samples showed higher saturated fatty acid and a lower monounsaturated fatty acid content with respect to semi-intensive ones. This appears to be the only significant difference, from the point of view of the nutritional value, found for the fishes reared with the two commercial systems investigated, semi-intensive and land based tanks intensive. Further analysis of samples, possibly taken during all the trial period would be needed to explain these data, or reveal additional differences and/or correlations [30].

## 3. Experimental Section

### 3.1. Sample Preparation andEnzyme Activity Assay

The trial was conducted in a time window between May and November, in Apulia Region (Italy) and three different samplings were carried out. Commercial extruded feed (Biomar-Treviso, Italy) was used for all fishes at a feeding rate of 1–2% fish body weight per day, 7 days a week. Final fish rearing density was 4, 15 and 25 kg/m^3^ for semi-intensive, sea cages and land-based intensive system, respectively. Enzyme activities were measured in the intestine and liver homogenate, according to Storelli *et al*. [31], using spectrophotometric techniques.

### 3.2. NMR Measurements

Lipid fractions, derived from muscle tissue of *Sparus aurata*, were extracted using Bligh & Dyer method [32] and analyzed by 1D and 2D NMR spectroscopy. NMR samples were prepared by drying the extract under a nitrogen stream, and subsequently dissolved in 1 mL of CDCl_3_. NMR spectra were obtained at 301.15 K on a Bruker Avance DPX 400 MHz. CDCl_3_ was used as solvent and chemical shift was referenced to TMS by the residual protic solvent peaks as internal references (δ_Η_ = 7.24 ppm; δ_C_ = 77.0 ppm).

^1^H-NMR spectra were acquired at 400.13 MHz of frequency, pulse program zg, 32K data points, spectral width of 11990 Hz, 128 scans with a 3 s repetition delay. ^13^C{^1^H} NMR spectra were acquired according to the following parameters: pulse program zgig30, spectral width 99911 Hz, 256K data points, 2.5 s repetition delay, 4 dummy scans, 6000 scans. Spectral processing, integration and deconvolution were performed using the software Topspin 1.2 (Bruker Biospin). Resonances of fatty acids were assigned on the basis of literature data [17,18,19,20,21,22].

## 4. Conclusions

The results obtained show that sea breams reared under specific conditions, both in a semi-intensive and intensive (land based tanks and sea cages) systems, present at the end of the breeding trial, a nutritional physiological state comparable to that of wild sea breams captured in a coastal area (control). The measured activity of the intestinal enzymes was similar in all samples, regardless of the source (semi-intensive, land based tanks intensive, sea cages intensive and wild type).

For semi-intensive and land based tanks intensive systems, the composition of the lipid fraction extracted from the fishes flesh was obtained by ^13^C-NMR and the lipid profile analyzed. No significant differences were observed for PUFA (DHA, EPA, 18:3) percentages for the samples coming from the two rearing systems examined [33,34,35]. On the other hand, small but significant differences were found in the monounsaturated/saturated fatty acid ratio, with the semi-intensive characterized by higher monounsaturated and lower saturated fatty acid content with respect to land based tanks intensive rearing system. Therefore the reported data suggest that, under the experimental conditions used, the monounsaturated/saturated fatty acid ratio is an observable parameter accounting for nutritional quality differences in lipid content of sea breams *S. Aurata* raised in the intensive and semi-intensive rearing systems.
